# Theoretical Analysis of an Optical Accelerometer Based on Resonant Optical Tunneling Effect

**DOI:** 10.3390/s17020389

**Published:** 2017-02-17

**Authors:** Aoqun Jian, Chongguang Wei, Lifang Guo, Jie Hu, Jun Tang, Jun Liu, Xuming Zhang, Shengbo Sang

**Affiliations:** 1 MicroNano System Research Center, Taiyuan University of Technology, Taiyuan 030024, China; jianaoqun@tyut.edu.cn (A.J.); tyut_wcg@163.com (C.W.); hujie@tyut.edu.cn (J.H.); 2 Key Laboratory of Advanced Transducers and Intelligent Control System, Shanxi Province and Ministry of Education, Taiyuan 030024, China; 3 Laboratory of Wireless Communication Network, Taiyuan University of Technology, Taiyuan 030024, China; guolifang@tyut.edu.cn; 4 State Key Laboratory of Science and Technology on Electronic Test & Measurement, North University of China, Taiyuan 030051, China; tangjun@nuc.edu.cn (J.T.); liujun@nuc.edu.cn (J.L.); 5 Key Laboratory of Instrumentation Science & Dynamic Measurement, North University of China, Ministry of Education, Taiyuan 030051, China; 6 Department of Applied Physics, Hong Kong Polytechnic University, Hung Hom, Kowloon, Hong Kong, China

**Keywords:** accelerometer, ROTE, finite element modeling, sensitivity, bandwidth

## Abstract

Acceleration is a significant parameter for monitoring the status of a given objects. This paper presents a novel linear acceleration sensor that functions via a unique physical mechanism, the resonant optical tunneling effect (ROTE). The accelerometer consists of a fixed frame, two elastic cantilevers, and a major cylindrical mass comprised of a resonant cavity that is separated by two air tunneling gaps in the middle. The performance of the proposed sensor was analyzed with a simplified mathematical model, and simulated using finite element modeling. The simulation results showed that the optical Q factor and the sensitivity of the accelerometer reach up to 8.857 × 10^7^ and 9 pm/g, respectively. The linear measurement range of the device is ±130 g. The work bandwidth obtained is located in 10–1500 Hz. The results of this study provide useful guidelines to improve measurement range and resolution of integrated optical acceleration sensors.

## 1. Introduction

Acceleration is a crucial parameter for monitoring the status of moving objects. Accelerometers have been a popular research object in recent years as they are widely used in many fields, such as aerospace, vibration sensing, and medical science [1,2]. Rapid advancements in microelectromechanical systems (MEMS) technology have allowed developers to fabricate acceleration sensors with miniaturized volume, low cost, and high sensitivity. The integrated optical acceleration sensor is a particularly notable example: this type of sensor is fabricated by utilizing fibers, optical gratings, and other separated optical elements, and typically has low integrated level and large volume [3,4]. The working principle of an optical accelerometer is typically based on FP cavity [5], waveguide coupling [6,7] and fiber Bragg gratings (FBG) [8,9]. Recent years have seen extensive research on the micro-size optical accelerometers, which were designed in an effort to improve the sensitivity and reduce the volume of the traditional accelerometers [10,11,12]. 

In this paper, we propose a novel optical acceleration sensor design based on the resonant optical tunneling effect (ROTE) [13]. The device’s structure, working principle, and performance will be discussed in detail below. As the successful direct displacement measurement is a key factor in acceleration evaluation, an extreme displacement resolution results in a high acceleration sensitivity; this is realized in the proposed accelerometer based on a unique physical structure of ROTE. When the sensor is under in-plane vibration (i.e., acceleration changes), the small displacement caused by the change in acceleration can be accurately measured by monitoring the shift of the resonant peak with ultrahigh optical quality factor Q (the value of which can up to 10^7^~10^9^). The ultrahigh quality factor Q meanwhile ensures excellent performance of the sensor.

## 2. Concept and Device Design

**Figure 1:**
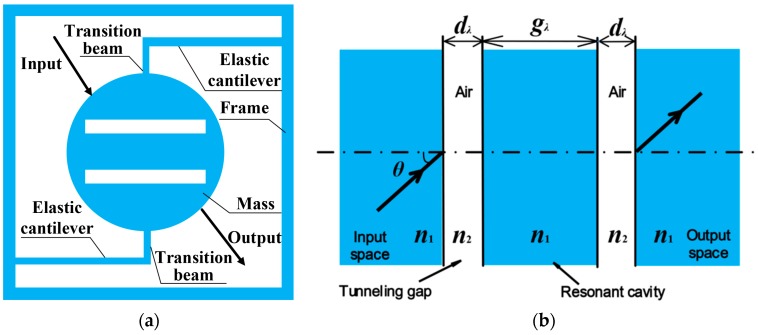
Device structure design and schematic diagram of the acceleration sensor. (**a**) Top diagram of the accelerometer structure; (**b**) Optical model of the ROTE structure.

Figure 1a shows a schematic diagram of the optical acceleration sensor, which consists of a fixed frame, two elastic cantilevers connected to the frame, and a central cylindrical mass comprised of a resonant cavity formed by two air tunneling gaps. The geometrical dimensions of the accelerometer structure are listed in Table 1. The radius and thickness of mass are 0.775 mm and 0.1 mm, respectively. The air tunneling gaps in the mass are ignored during the FEM simulation because their total width is only 4.557 μm, comprising only 0.375% of the whole mass (The length of the air tunneling gap is considered to be 1.55 mm for the purposes of calculation). 

The central mass acts as the sensing element, which is in the form of a typical ROTE structure. The device can be made of silicon wafer and fabricated using standard silicon bulk micromachining technique. Micromachined hemicycle silicon prisms connected with slim cantilevers can be formed using deep reactive ion etching (DRIE) with high dimensional accuracy. The roughness of the sidewall can be greatly eliminated by reducing the etching passivation period (the optimal *R_q_* = 20 nm can be obtained in SPTS LPX DSi), and be further diminished by several methods (*R_q_* = 1~2 nm), such as thermal oxidation [14], laser reformation technique [15], and hydrogen annealing [16,17]. Some similar structures have been fabricated successfully by the authors and applied for thermal-optic switches in previous work [18,19]. Unlike the traditional piezoelectric accelerometer, which detects the acceleration perpendicular to the sensor structure plane, the ROTE sensor can measure the acceleration in the plane where the device is placed. Once an external acceleration is applied, taking the upward acceleration for example, the mass will produce the inverse displacement (downward) due to its inertia. The incident light thus hits different points on the central mass to produce different incident angles. 

As a unique mechanism, ROTE has been experimentally demonstrated by two previous research teams [20,21] and proposed theoretically for use in optical switches [18] and refractive index (RI) sensors [22]. Figure 1b depicts the optical ROTE structure model containing five layers in total: three high RI layers are sandwiched (Si, in dark color) with two low RI layers (air, in light color). The initial angle of incident light will be higher than the critical angle on the interface of the input medium and the air tunneling layer. The incident light tunnels across the air layer and propagates through the resonant cavity before passing another tunneling layer and reaching the output prism, where it forms the transmission output. The design parameters of the ROTE structure are listed in Table 2. The widths of the resonant cavity and dual tunneling gaps are all normalized to the wavelength of the incident light. Once the sensor is subject to the external acceleration, the displacement of the mass causes a change of the incident point on the sidewall of the cylindrical mass, resulting in a variation of the refracted angle after the cylindrical air/mass interface and thus a change of the incident angle to the first air tunneling gap. Finally, the P-polarized or S-polarized light transmitted through the central mass will present a shift of the transmission wavelength, which is monitored by the photodetector. 

## 3. Theoretical Analysis and Simulation

The ROTE optical acceleration sensor uses a wide spectrum light source to monitor the shift of the transmission peak. Below, this part will first present our investigation of the change in the incident angle along with the displacement of the central mass; next, the transfer matrix method (TMM) is utilized to analyze the ROTE structure in regards to the output spectrum with respect to the incident angle. A detailed description of TMM can be found in the authors review paper [13]. The theoretical relationship between the displacement and the light transmission is established accordingly. The simulation model of the optical accelerometer will also be discussed and used to study the mechanical characteristics of the proposed structure. Finally, the performance of the sensor is discussed based on the simulation results above and compared against similar Fabry-Pérot (FP) accelerometers in terms of their respective performance parameters.

### 3.1. Derivation Formula of Linear Displacement from the Incident Angle 

This part will study the dependence of the transmission on the linear displacement. According to the design, the direction of the incident light source is fixed as *β* degree to the horizontal direction. The designated parameters of the ROTE structure are listed in Table 2.

**Figure 2:**
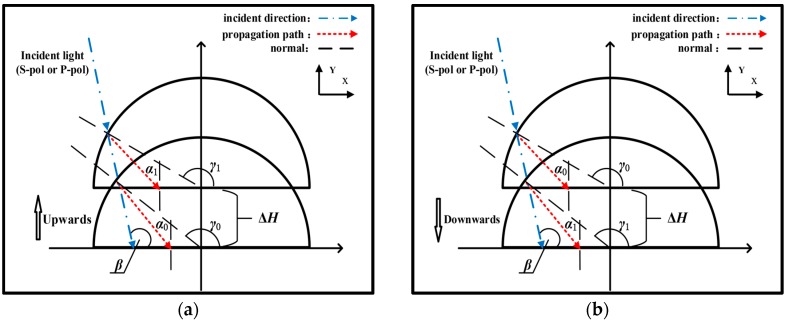
Change of displacement Δ*H* under perpendicular vibration. (**a**) Upward; (**b**) Downward.

When the sensor is under an in-plane acceleration, the mass is translated in the opposite direction due to the inertia. Vertical acceleration, for example, results in the relative movements of the mass with respect to the frame shown in Figure 2a (upward) and Figure 2b (downward), respectively. As shown in Figure 2a, after the refraction on the mass surface, the S-polarization (or P-polarization) light arrives at the interface of the input space and the first tunneling gap at an initial value of *α*_0_. Under the upward linear acceleration, the displacement of the mass is Δ*H* and the incident angle *α*_0_ is changed to *α*_1_. The situation is similar when the acceleration is downward (Figure 2b). According to the geometric relations, the displacement Δ*H* in Figure 2a,b can be written as follows

(1) $$\Delta H=R\cdot \left(\sin\gamma_{0}-\sin\gamma_{1}\right)+R\cdot \tan\left(\pi -\beta \right)\cdot \left(\cos\gamma_{0}-\cos\gamma_{1}\right)$$

where *R* is the radius of the mass (775 μm); *γ*_0_ and *γ*_1_ are the angles between the corresponding normal line of incident light and the *X*-axis. The incident angle *α* and angle *γ* have the following relationship,

(2) $$\alpha_{0}=\gamma_{0}-\frac{\pi }{2}-\arcsin\left(\frac{\sin\left(\gamma_{0}-\beta \right)}{n_{si}}\right)$$

(3) $$\alpha_{1}=\gamma_{1}-\frac{\pi }{2}-\arcsin\left(\frac{\sin\left(\gamma_{1}-\beta \right)}{n_{si}}\right)$$

In Figure 2, angle *β* is fixed as 105 degrees. The static incident angle is 22.5884 degrees and the designed measuring range is ±0.1 degrees. For the incident angle in the measuring range, variations in transmission intensity can be neglected according to the Fresnel formula. (i.e., for P-polarization, the normalized transmission *T* is 0.6951 for the static incident angle *α*_0_ = 22.5884 degrees but it becomes 0.6950 for the maximum incident angle *α*_0_ = 22.6884 degrees). Accordingly, the difference in the transmission power caused by the refraction occurring on the incident surface of the mass can also be ignored. It is important to note that the mass will produce a lateral displacement in the in-plane acceleration. This lateral displacement phenomenon, as discussed below, will be analyzed in the simulation.

### 3.2. Spectrum-Based Displacement Measurement Based on ROTE

The spectrum-based displacement analysis employs a broadband light source as the input, then the results are obtained by observing the transmission peak in the output spectrum. This approach reduces the design requirements and does not require a strict control of experimental conditions (e.g., initial angle of the incident light source), making it readily and feasibly implementable. The broadband light source can self-compensate for fabrication error and drift of experimental conditions by relocating the transmission peak. This setup also makes experimental observation easier, as there is always a peak in the output spectrum.

**Figure 3:**
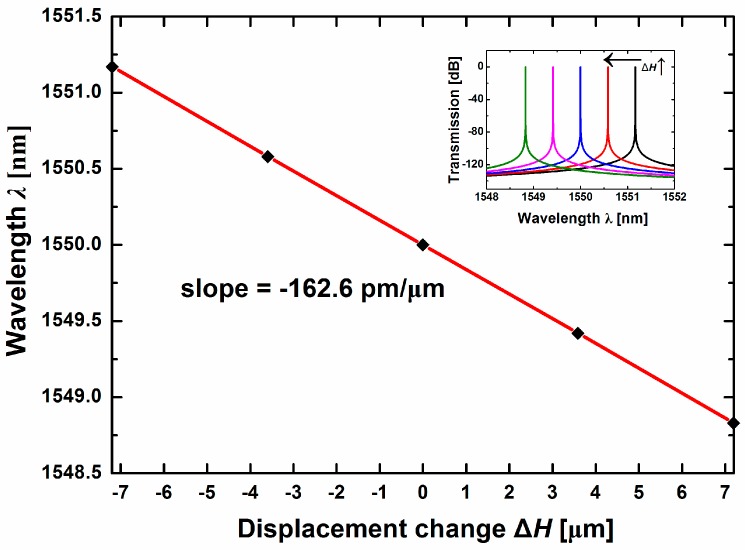
Shift of the transmission peak wavelength with respect to the displacement Δ*H* caused by the acceleration. The inset exemplifies the transmission peaks and their shift with the increase of Δ*H*.

Assuming the S-polarization incident light has a central wavelength of 1550 nm and a bandwidth of 4 nm, the dependence of the peak shift on the displacement Δ*H* as plotted in Figure 3. To limit the measurement error caused by lateral displacement in an acceptable range (2%), the designed incident angle measurement range is ±0.1 degrees, thus the linear displacement measurement range of Δ*H* is ±7.194 μm according to Formulas (1)–(3). As seen in Figure 3, when the sensor is static, the relative displacement of the mass Δ*H* is zero and the wavelength of ROTE transmission peak is 1550 nm. Once an upward acceleration is applied, then a positive displacement takes place, and the wavelength of the ROTE peak will present a blue shift. The wavelength will fall to 1548.83 nm when the maximum displacement Δ*H* = 7.194 μm. On the contrary, the ROTE peak produces a red shift in case of the applied acceleration in the opposite direction. The reason behind the phenomenon is, as the incident angle changes with the applied acceleration, the wavelength of the transmission peak should in turn shift because the corresponding wave vector conforming to the inherent transmission mode of the ROTE structure remains stable. The inset shows the transmission spectra corresponding to different Δ*H* values. An increase in the displacement Δ*H* causes a blue shift of the ROTE peak, and such shift is highly linear. The slope between the wavelength *λ* and displacement Δ*H* obtained is −162.6 pm/μm. 

**Figure 4:**
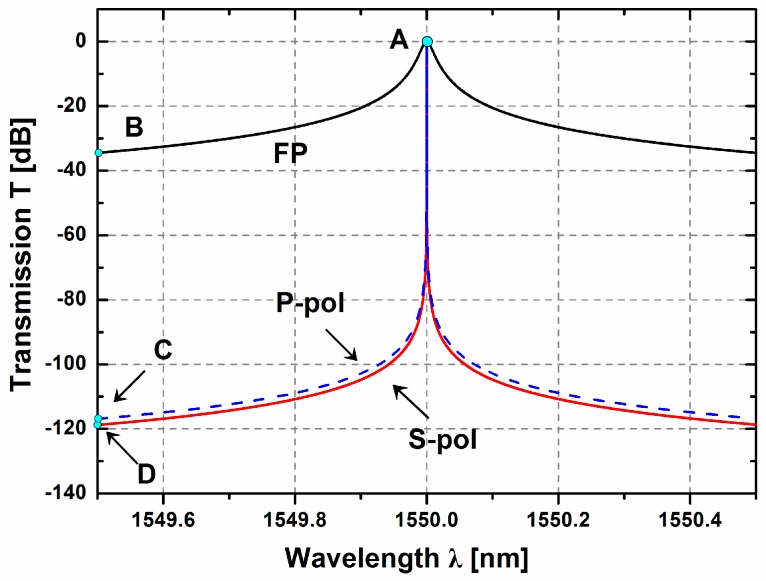
Transmission curves of both S- and P-polarizations with respect to displacement Δ*H*. For comparison, the transmission of the FP etalon (*R* = 0.999, cavity length 12.94*λ*) is also plotted as a reference.

Figure 4 indicates the transmission contours of both S- and P-polarizations with respect to the wavelength *λ*. The initial transmission *T* is located at point A (for S-polarization or P-polarization) under the static condition. By comparing the two curves of S-polarization and P-polarization, it is clear that the S-polarization transmission experiences a faster drop than that of P-polarization. For the P-polarization, *T* drops from 0 dB (point A) to −116.85 dB (point C) when the wavelength change is Δ*λ* = 0.5 nm; while for the S-polarization, it drops to −118.77 dB (point D). Based on this observation, the transmission *T* is more sensitive for the S-polarization light. In the case of S-polarization, the quality factor Q is also much higher than that with the P-polarization—up to 8.857 × 10^7^ based on our calculation results.

The FP etalon is known for its high sensitivity of wavelength selection and is often used as a reference for spectral detection [5]. The classic FP etalon usually utilizes the change of the physical length of the etalon for accelerate sensing. In order to fully demonstrate the ultrahigh sensitivity of the proposed optical sensor, the transmission variation of the FP etalon with the same refraction mirrors is also illustrated in Figure 4. It is assumed that the mirrors have 99.9% reflectivity and the initial incident angle of the FP cavity is 0 degrees. According to Table 1, the length of the FP resonant cavity is 12.94*λ* (i.e., 20.057 μm for *λ* = 1.55 μm), essentially the same as the effective length of the ROTE region (i.e., resonant cavity width + 2 × tunneling gap width). For Δ*λ* = 0.5 nm, the transmission of FP only drops from 0 dB to −34.47 dB (point B). This transmission drop of FP is about three orders lower than the drop of −118.77 dB in the acceleration sensor. The Q factor of FP is 8.183 × 10^4^, which is three orders lower than that of S-polarization (i.e., 8.857 × 10^7^). Thus, it is reasonable to expect that the ROTE sensor can achieve a resolution about three orders of magnitude higher than the FP sensor. The reason behind this phenomenon is that the ROTE structure takes advantage of the evanescent wave originated from the total reflection effect to form a reflection interface with ultrahigh reflectivity, which comprises the resonant cavity in the middle of the system.

The FP etalon or P-pol also produces the peak shift in response to the displacement Δ*H*. However, their characteristic peaks are broader than that of S-pol and thus it makes it difficult to locate the exact maximum point of the peaks (especially in the environment of strong noise). Thus, the S-polarization light is certainly preferable as the light source in the proposed design.

**Figure 5:**
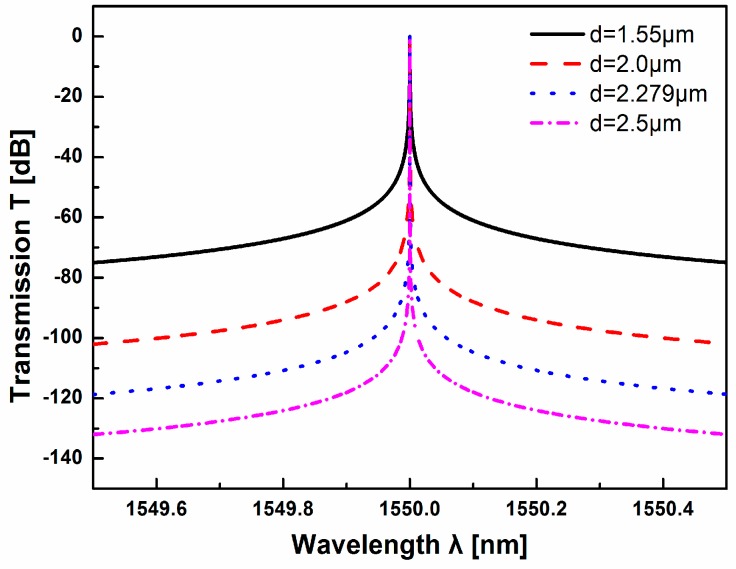
Transmission curves of S-polarization with respect to tunneling gap *d*.

In case of the ROTE structure, the dependence of the transmission *T* in S-polarization on the tunneling gap *d* is shown in Figure 5. With the increase of the air gap *d*, the Q factor of ROTE structure is higher (from 8.76 × 10^6^ to 1.41 × 10^8^). But the position of the resonance peak does not change during the increase of the tunneling gap *d*, which is convenient for observing the transmission peak in the output spectrum. For spectrum-based sensing methods, a large spectral width makes it hard to find the accurate position of peak (or dip), thus the large Q value is conducive to high resolution sensing. However, considering the cost and complexity of the system, if the *d* value (1.61*λ*, 2.5 μm) is too large, the transmission peak is too sharp to be observed easily, so the medium *d* value (1.47*λ*, 2.279 μm) is chosen in the following analysis.

### 3.3. Simulation of ROTE Accelerometer Model

This section will discuss the design of the mechanical structure of the acceleration sensor. The mechanical characteristics of the proposed structure are investigated via COMSOL Multiphysics with the finite element method (FEM).

**Figure 6:**
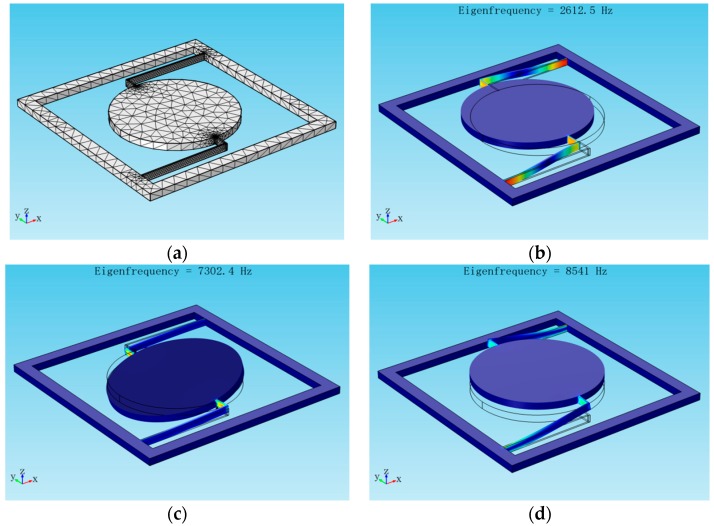
Model simulation of proposed accelerometer via FEM. (**a**) Model structure; (**b**) The first mode; (**c**) The second mode; (**d**) The third mode.

In the FEM simulation, the square frame is added as a fixed constraint when solving the resonant frequency. The cylindrical mass and elastic cantilevers can be moved freely. According to the design parameters of the sensor structure listed in Table 1, Figure 6 shows the model structure and first three-order vibration modes of the accelerometer, where the resonant frequencies are 2613 Hz, 7302 Hz, and 8541 Hz. The first resonance mode (Figure 6b) causing peak wavelength shifts is related to the movements of the cylindrical mass along the sensing *Y*-axis, which is selected as the working mode of the device. The second (Figure 6c) and the third (Figure 6d) mode refer to, respectively, the rotational mode (rotary along the *Y*-axis) and vertical mode (vertical vibration in the *Z*-axis) of the sensor. However, as the second and the third resonant frequency (frequency = 7302 Hz, 8541 Hz) of the structure are much larger than that of the first mode, they will not affect the proposed accelerometer operation when the working frequency is much lower than the eigenfrequency of the first mode. 

The frequency of the first mode *f* also can be deduced by calculating the resonant frequency of the mechanical structure in the lumped model

(4) $$f=\frac{1}{2\pi }\times \sqrt{\frac{k_{sys}}{m}}$$

where *m* is the mass of the proof mass and *k_sys_* is the total spring constant along the *Y*-axis. In case of the crab-leg flexural beam, the total spring constant *k_sys_* of can be found as [23,24]

(5) $$k_{sys}=\frac{6EI_{E}(I_{E}L_{T}+4I_{T}L_{E})}{{L_{E}}^{3}(I_{E}L_{T}+I_{T}L_{E})}$$

with

(6) $$I_{E}=\frac{1}{12}H_{E}{W_{E}}^{3},I_{T}=\frac{1}{12}H_{T}{W_{T}}^{3}$$

where *E* is the young’s modulus of silicon, *I_E_*, *L_E_*, *H_E_*, *W_E_*, are the moment of inertia, length, height, and width of the elastic cantilever and *I_T_*, *L_T_*, *H_E_*, *W_T_*, are the moment of inertia, length, height, and width of the transition beam. As a result, the resonant frequency obtained by calculation is 2930 Hz with the total spring constant *k_sys_* 153 N/m (young’s modulus = 170 GPa) and the mass *m* 4.5218 × 10^−^^7^ kg (density *ρ =* 2329 kg/m^3^). The acceptable difference (10%) between the values derived from the theoretical calculation and FEM simulation (2613 Hz) originates from the differences in assumptions and models of these two methods. 

**Figure 7:**
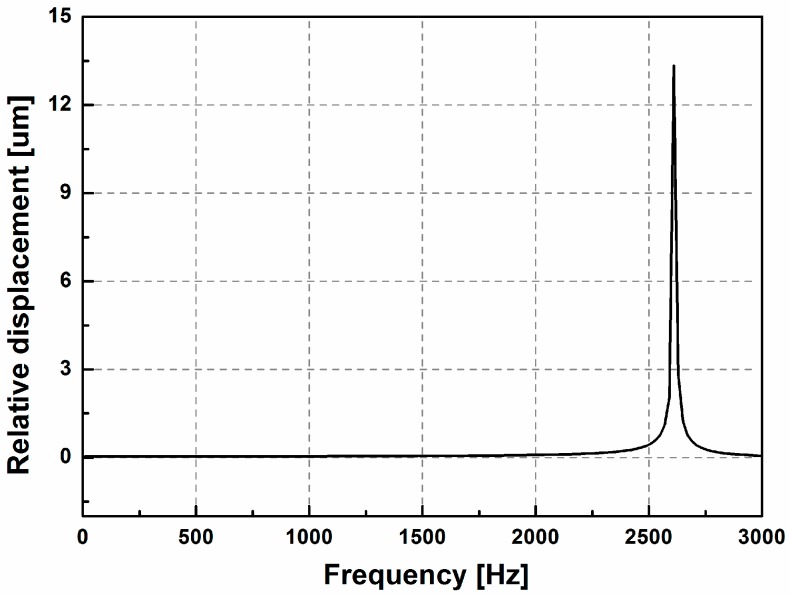
Frequency response curve of the optical sensor.

In addition to the vibrational model, the working bandwidth is also a key parameter of an optical sensor. The eigenfrequency of the proposed accelerometer is studied, as the working frequency of the accelerometer must be far from its eigenfrequency to protect the device from damaging resonance. Assuming a 1*g* (*g* = 10 m/s^2^) acceleration is applied to the square frame in the direction parallel to the *Y*-axis (the sweep frequency ranges from 10 to 3000 Hz), the relative displacement as a function of the frequency of applied acceleration is plotted in Figure 7. The work bandwidth of 10~1500 Hz is selected because the frequency response curve is relatively flat over this range.

To optimize the performance of the sensor, the dimensions of the elastic beam (including the elastic cantilever and the transition beam in Figure 1a) are analyzed. Since the length of the elastic cantilever is as long as half length of the square frame, which lies on the volume of the accelerometer. Therefore, its value is fixed in the following simulations. 

**Figure 8:**
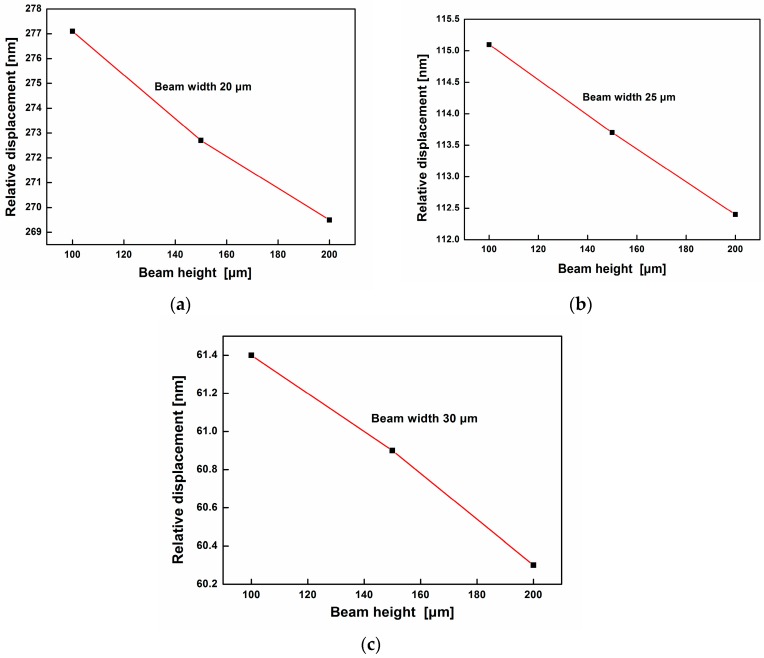
Relative displacement curves with respect to the elastic beam height in different beam widths (**a**) 20 μm; (**b**) 25 μm; (**c**) 30 μm.

The width and height of the elastic beam are investigated to achieve a higher sensitivity of the accelerometer. Assuming a 5*g* (*g* = 10 m/s^2^) acceleration excitation signal is applied to the square frame in the designed direction parallel to the Y-axis with a frequency of 1500 Hz, the relative displacement contours with respect to different width and height values are shown in Figure 8. It can be observed that the relative displacement decreases with the increase of the cantilever height when the beam width is constant. By comparing with the Figure 8a–c, the relative displacement also drops with the rise of the cantilever height. As a result, when the cantilever height and width are 100 μm and 20 μm, respectively, the relative displacement can reach the maximum value 277 nm, so the design with this set of dimensions has the maximum sensitivity based on the simulation results and is adopted as an example in following analysis. However, the measurement range of acceleration will be reduced at the same time due to the limit of the displacement of mass (7.194 μm). It means the accelerometer is more sensitive to the change of the acceleration despite the reduction of measurement range. Therefore, the design of the sensor should balance both the sensitivity and the measurement range according to the requirements of specific application. 

**Figure 9:**
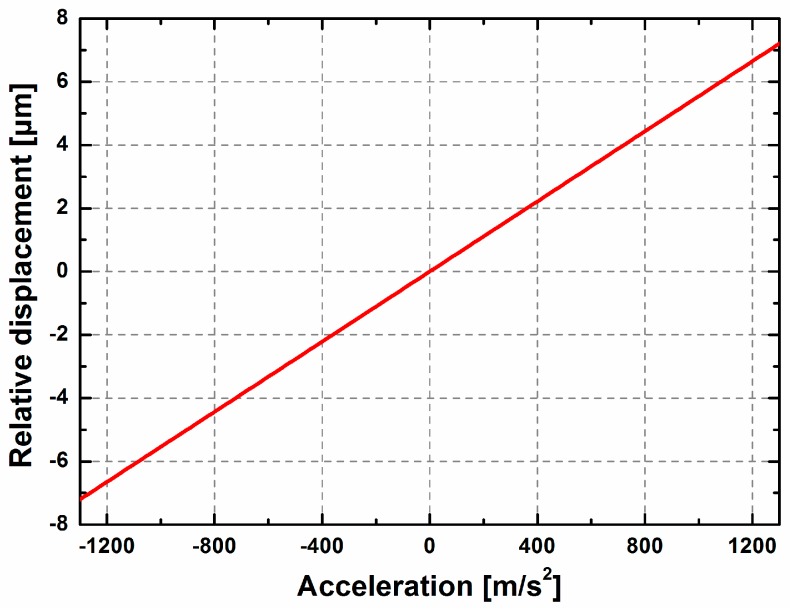
Relationship between the relative displacement and the applied acceleration.

The following simulation is performed to further elucidate the sensitivity of the proposed sensor. Because the cantilever is more easily bent under high working frequencies, a higher frequency excitation signal of 1500 Hz is chosen as the working frequency so as to test whether the cantilever beam can work beyond the bending strength. The sweep ranging from −1300 m/s^2^ to +1300 m/s^2^ is applied to the square frame, and the boundary constraints are as same as that in the simulations above. The relative displacements under different external accelerations are exhibited in Figure 9. Based on these simulation results, the maximum evaluated acceleration of 1300 m/s^2^ (the corresponding maximum longitudinal displacement is 7.194 μm, of which the corresponding bending strength is 42.9 MPa in the root of cantilever) is detectable before the elastic cantilever reaches the maximum bending strength of 80 MPa for silicon. This proves that the accelerometer responds normally in the dynamic range of the measurable acceleration. The mass produces a lateral translation of 0.158 μm in this case, which is negligible, because it comprises only 2% of the maximum longitudinal displacement (7.194 μm). 

**Figure 10:**
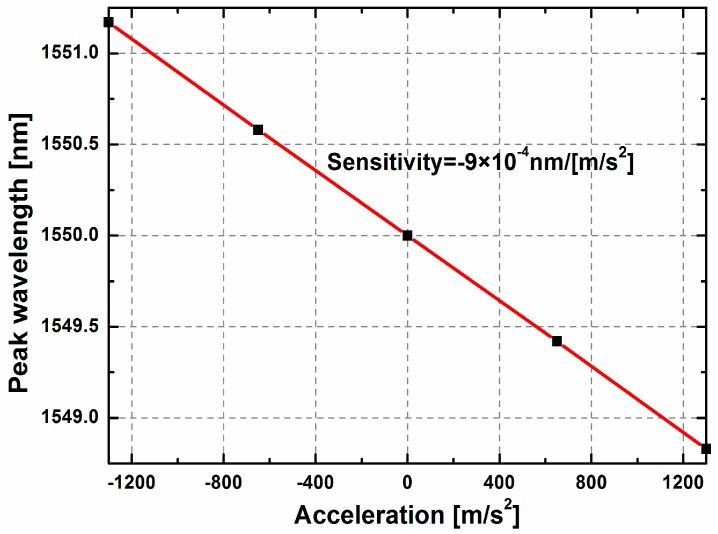
Relationship between the peak wavelength of ROTE transmission and the applied acceleration.

The transmission *T* for the S-polarization as a function of the applied acceleration is plotted in Figure 10. The sensitivity for the in-plane accelerometer is 9 × 10^−4^ nm/(m/s^2^) (i.e., 9 pm/g) based on the linear fitting, with the standard error of 1.776 × 10^−6^ nm/(m/s^2^). With a high-resolution optical spectrum analyzer (0.01 picometer), the resolution of the ROTE senor can reach 1 × 10^−3^ g. Although the sensitivity is a key parameter for the accelerometer, the sensitivity and the acceleration measurement range always should be balanced for the specific application. According to the simulation results, the proposed accelerometer can reach 9 pm/g with measurement range of ±130 g, which is comparable with that of the Fabry-Pérot-based accelerometer (90 nm/g, ±0.263 g) [5], the photonic crystal accelerometer (1.17 nm/g, ±22 g) [25] and the optical microring resonator accelerometer (31 pm/g, ±7 g) [26]. The sensor we proposed has the lowest sensitivity but maximum measurement range among all these optical accelerometers. Furthermore, the ultrahigh optical Q value (8.857 × 10^7^) of the characteristic peak is much higher than that of the optical microring resonator (1 × 10^5^) [26] and the photonic crystal optomechanical cavity (7 × 10^6^) [27], which promises a higher resolution for the spectrum-based sensing method. This is a great advantage in application.

Although the present study mainly focuses on the theoretical analysis, the experiment will better reveal the performance of the sensor. In order to realize the test analysis, the broadband light source (including optical fiber based collimator and polarization control) and photodetector will be fixed in the silicon structure fabricated in advance. The ROTE transmission detected by photodetector at the output side will be transferred into voltage signal. Furthermore, the static and dynamic characterizations of the accelerometer (including the sensitivity, resolution, dynamic range, etc.) will be carried out by using the test system of a rotating table and vibration shaker. 

## 4. Conclusions

In this study, we present a new design of optical accelerometer by exploiting the special physical mechanism of the resonant optical tunneling effect. The structure and sensing principles of the sensor are studied in detail, and the mechanistic characteristics of the proposed structure are analyzed via finite element method. Theoretically, the proposed ROTE-based accelerometer performs very well. The transmission peak has a Q factor is about 1 × 10^3^ times higher than the traditional Fabry-Pérot cavity. The in-plane acceleration with the bandwidth of 10~1500 Hz can be detected by the optical accelerometer with the sensitivity of 9 pm/g and the linear measurement range of ±130 g. The accelerometer can be applied in high precision inertial systems and other similar applications.

## Figures and Tables

**Table 1:** Partial design parameters of the sensor structure (μm).

Parameter	Length	Width	Height
Square frame	2650	300	100
Elastic cantilever	1165	20	100
Transition beam	200	20	100

**Table 2:** Design parameters of the ROTE structure.

Parameter	Symbol	Values	
		*s*-Pol	*p*-Pol
Static incident angle	*θ*	22.5884°	22.5884°
Normalized width of the tunneling gap	*d*_λ_ *	1.4700	1.4700
Normalized width of the resonant cavity	*g*_λ_ *	10.0023	10.0790
Refractive index of input and output space	*n*_1_	3.420	3.420
Refractive index of the resonant cavity	*n*_1_	3.420	3.420
Refractive index of the air tunneling gap	*n*_2_	1.000	1.000

* *d*_λ_ and *g*_λ_ are all normalized to the wavelength of incident light.
